# The Literary Side of Our Profession

**Published:** 1896-05

**Authors:** Wm. H. Steele

**Affiliations:** Forest City, Iowa


### The Literary Side of our Profession.

RY DR. WM. H. STEELE, FOREST CITY, IOWA.

Read before the Iowa State Dental Society at Marshalltown, May, 1896.

*Mr. President and Gentlemen:*

In every department of life there was a time when a young man was only educated in those branches which were absolutely necessary for him in the sphere in which he lived, or for the occupation or profession which he expected to follow through life. Those who had but little education themselves knew but little of its advantages or importance, and, of course, considered all education not absolutely required for every-day use a needless luxury.

A young man was thus confined to the narrow limits of his own immediate duties, and seldom had the means, opportunity or ambition to break away from this beaten path and rise to higher and broader fields of usefulness.

This was true of dentistry in its earlier days; young men only acquired sufficient knowledge of the business to enable them to practice it in a mechanical sort of a way. Some who were mechanical geniuses became skillful manipulators, but the larger number never rose above the rote method taught them by their preceptors, and had no higher aspirations. But this has materially changed. The almost universal education of children in our public schools lays the foundation of a desire to study, so that, now, with our myriads of good, low-priced literature and numerous public libraries but few come to maturity without a good stock of general knowledge, and it is to one's own discredit if he allow his literary talents to fossilize at this point in life.

When a young man enters upon active practice he should make it a fixed habit to continue his course of reading, but sad to say, with too many of us, all literary work is cut off short, right where active business life begins. How true and full of wisdom are the words of Lord Bacon: "Reading makes the full man; speaking makes the ready man, and writing makes the exact man." The first and last of these are full of wise instruction to the dentist, but we are compelled to confess among ourselves that these duties are shamefully neglected by a large majority of our profession.

The young man usually starts out with good resolves, and reads systematically until his professional duties or other business matters begin to encroach upon his reading hour, then he finds plenty of plausible excuses for its neglect; gradually his taste and fondness for reading is lost and the work becomes irksome. He may take occasional spurts and read up on some case in practice, but, sad to say, even that ceases; he drops into the "back number rut," and instead of the "full man," he becomes lean and rusty in all that is new in dental literature. Ask him now to write a paper for the society or an article for the journal and he will reply: "I can't write, I have enough ideas but cannot express them on paper." Quite true; neither can a good crop be produced from an uncultivated field. Reading is the great cultivator of the human mind which prepares it for literary production.

It is impossible for any one to be a good writer without being a reader, and one who is a systematic and thorough reader only requires *practice* to become a writer. We would like to emphasize the third part of Lord Bacon's saying: "Writing makes the exact man," and it can be easily shown why. When one goes about writing on any subject he first makes some arrangement in his own mind. We call this getting out the frame timber, and it must be done with care and exactness in order to make a complete structure, and if the subject is of importance the more pains will be taken in its preparation.

Whilst arranging his thoughts he will be trying to inform himself, for now he begins to see that he don't know as much as he thought he did. The dentist will go to his books or among his file of dusty journals, having an indistinct recollection of something there that has a bearing on the subject; begins to cull a thought here and there and discards a preconceived notion for something better. All this is cultivating the mind and good training, for he is learning to discriminate and is gathering up facts enough to amply repay him for all time and labor. His writing is intended for publication, or to be read at the society meeting and must stand the test of criticism. He is fully aware that that test is a severe one and that no loose or random thought must find expression; every position must be fortified and protected; every declaration must admit of verification and the result is the utmost exactness of which he is capable. The writer is benefited whether a new thought is advanced or not, and the mental training is profitable to himself whether it is to any one else or not. Whilst exactness of thought in our profession is to be sought after it should not end with the person himself, as an unborn thought is of but little good to the world; the reader and thinker should cultivate the faculty of arranging and imparting his thoughts to others who are to come after him. How often in our travels, or at society meetings we have met men of twenty or thirty years practice who are fine operators, have inventive talent and are highly instructive in private conversation yet can not write a readable article. They have never been trained in that school of exactness which comes of writing, and the benefits of a long life of experience must be lost to the profession.

Our dental journals offer good training ground for the young writer, and every young practitioner should avail himself of the advantages offered (for unless we begin writing when young, unfortunately we will not take to it when old), and contribute one or two articles a year upon some favorite subject which he is willing to take the time to investigate. As before said, he will be well compensated for his time and labor. To be *sure* he may have one or two of his first articles returned or thrown into the waste basket, but this should not discourage further efforts, and should only stimulate him to renewed and increased exertion.

When a man makes up his mind to write, his professional life assumes a new phase; the dogged tread-mill of every-day sameness is gone; he has something to think of while pursuing his daily routine; he takes a new interest in looking for new features; his powers of observation and classification are receiving cultivation and his mind will grow and expand like a well-watered plant in the summer sunshine— a reflex action of the framing and building process illustrating the old poverb, "It is more blessed to give than to receive."

Many who are located in small country towns think they can not furnish a creditable paper or worthy clinic for their society because they live in a small place and do not have the advantages of their brothers who live in the cities. We believe in the old adage, "Necessity is the mother of invention," and originality, if encouraged in its development, is to be found in the country more than elsewhere, as the country dentist is thrown more upon his own resources and has to think and act for himself in all sorts of emergencies.

Now, when you go home slip a little note-book into your vest pocket, sharpen up your pencils and begin your notes upon some subject that suits you; add a stick of timber here and there as you find it to the frame until the skeleton is complete; then clothe it from time to time as you have the leisure, and next year, when some member of the executive committee writes you for a paper, don't reply, "I can't write"—it is too lame an excuse; every one has some good thoughts that stagnate for want of expression, for, as the poet says: "Thoughts shut up, want air, and spoil like bales unopened to the sun."

